# SWI/SNF complex alterations predict immunotherapy response in bladder cancer

**DOI:** 10.3389/fimmu.2025.1708324

**Published:** 2025-12-08

**Authors:** Jian Zhang, Yapeng Wang, Qian Yan, Haitao Wang, Qiang Ran, Hailin Zhu, Weiming Luo, Yangkun Ao, Ying-ang Ji, Jing Xu, Jun Zhang, Yao Zhang, Jun Jiang, Qiuli Liu, Weihua Lan

**Affiliations:** Department of Urology, Daping Hospital, Army Medical University, Chongqing, China

**Keywords:** bladder cancer, immune checkpoint inhibitors, machine-learning, prediction model, SWI/SNF

## Abstract

**Background:**

Immune checkpoint inhibitors have revolutionized the treatment of urothelial bladder cancer (UBC), yet response remains limited to a subset of patients. The SWItch/sucrose non-fermentable (SWI/SNF) chromatin remodeling complex is recurrently altered across cancers, but its prevalence, functional impact, and predictive value in UBC remain unclear. This study aimed to comprehensively delineate the mutational spectrum of SWI/SNF genes in UBC and assess their utility as predictive biomarkers for response to immune checkpoint blockade.

**Methods:**

We analyzed tumor specimens from 49 patients in the Daping Cohort and integrated data from five independent public cohorts comprising 2,280 cases in total. Somatic alterations were identified using targeted or whole-exome sequencing, and transcriptomic profiles were obtained from RNA sequencing datasets. Survival outcomes were evaluated using Kaplan–Meier survival analysis and time-dependent ROC curves. Tumor immune landscape was characterized via xCell-based deconvolution and corroborated by multiplex immunofluorescence on institutional samples. Prognostic modeling was performed across 65 machine-learning configurations, encompassing survival SVM, CoxBoost, and stepwise Cox, with external validation in independent cohorts.

**Results:**

SWI/SNF alterations were present in 42.8% of UBCs, with the highest frequencies in *ARID1A, ARID1B, ARID2, SMARCA4*, and *PBRM1*. Tumors harboring these alterations displayed higher tumor mutational burden, increased neoantigen load, an immune-inflamed microenvironment, and a significantly improved overall survival following immune checkpoint blockade (*p* < 0.05). Genotype-specific models achieved strong prognostic discrimination (C-index > 0.75), with AUCs up to 0.909 in SWI/SNF-mutant and 0.772 in wild-type tumors, substantially outperforming single-modality biomarkers.

**Conclusions:**

SWI/SNF alterations define an immunotherapy-responsive stratification of UBC. The accompanying genotype-specific prognostic models provide a ready-to-test framework for guiding precision immunotherapy.

## 1 Introduction

Bladder cancer represents the fourth most prevalent malignancy among men worldwide, ranking ninth in global incidence and thirteenth in mortality (1). Pathologically, most cases are urothelial carcinoma, clinically stratified into non-muscle-invasive bladder cancer (NMIBC) and muscle-invasive bladder cancer (MIBC) disease based on depth of invasion. The past decade has witnessed a paradigm shift in the management of advanced bladder cancer with the administration of immune-checkpoint inhibitors (ICIs). Landmark trials including CheckMate-032 and NABUCCO have demonstrated substantial increase in objective response rates (ORRs), pathological complete response (pCR) rates, progression-free survival (PFS) and overall survival (OS) with immunotherapy (2–4). In the neoadjuvant or bladder-sparing setting, ICIs-based combination regimens are already redefining standard-of-care paradigms for MIBC (5, 6). Despite these advances, a fundamental limitation persists across solid tumors, including bladder cancer: only 20–30% of patients derive durable benefit from these agents (7). Established biomarkers such as PD-L1 expression and tumor mutational burden (TMB) (8) lack sufficient predictive power, largely attributable to extensive inter- and intra-tumoral heterogeneity (9). Consequently, identifying more reliable determinants of ICIs response represents an urgent unmet need to fully realize the potential of immunotherapy in this disease.

The SWItch/sucrose non-fermentable (SWI/SNF) chromatin-remodeling complex is an evolutionarily conserved, multi-subunit machinery comprising 29 core proteins that collectively orchestrate transcription, DNA replication, and the DNA-damage response (10, 11). In almost all types of tumor, at least one SWI/SNF subunit is somatically altered, with mutation frequencies often approaching 20% (12). While these mutations can function as potent drivers of malignant progression by enhancing proliferation, invasiveness, and metastatic potential, individual subunits often exert context-dependent, and sometimes opposing effects on tumor progression and therapeutic sensitivity. For instance, loss of *ARID1A* or *ARID1B* confers enhanced response to immune-checkpoint blockade (ICB) in melanoma and non-small cell lung cancer (13, 14), whereas *PBRM1* deficiency promotes primary resistance in renal cell carcinoma (15). Although sporadic reports have associated SWI/SNF alterations with higher grade, advanced stage, and increased invasiveness (10, 16, 17), a systematic analysis of the entire complex is lacking, and the functional consequences of specific subunit alterations, particularly with respect to their impact on immunotherapy outcomes of UBC, remain largely unexplored.

Building on these knowledge gaps, the present study undertakes a comprehensive molecular portrait of SWI/SNF complex alterations in bladder cancer. By integrating large-scale genomic, transcriptomic, and immunotherapy-response datasets, we systematically: (i) delineate the full mutational landscape of all SWI/SNF subunits; (ii) decipher the resultant mechanisms of tumor–immune interactions; and (iii) rigorously assess their predictive value for ICIs benefit. Establishing a robust, mutation-informed biomarker framework would not only optimize patient stratification for existing immunotherapies but also illuminate avenues for potential combination strategies, ultimately advancing precision immuno-oncology for this genomically heterogeneous disease.

## 2 Materials and methods

### 2.1 Patient cohorts and data acquisition

This study integrated data from a maintained institutional cohort together with multiple publicly available datasets. The primary discovery cohort consisted of 49 UBC patients from Daping Cohort. Detailed clinical and primary sequencing data are available under accession number HRA004401 (18), and the baseline characteristics of these patients are summarized in **Supplementary Table S1**. The current analysis specifically focused on systematic profiling of SWI/SNF complex gene mutations (29 core subunits) and multiplex immunofluorescence-based assessment of the immune microenvironment in 13 MIBC samples from this cohort. The study protocol was approved by the Ethics Committee of the Army Medical Center (Approval No.201896) with written consent obtained from all participants. All procedures involving human subjects were conducted in accordance with institutional ethical standards, the 1964 Declaration of Helsinki, and its subsequent amendments or comparable ethical guidelines. Targeted sequencing was performed using commercial platforms, covering panels of 87 genes (n = 3), 891 genes (n = 7), 1,054 genes (n = 19). Alternatively, whole-exome sequencing was performed for 20 samples.

To validate and extend our findings, we obtained extra data from four independent bladder cancer cohorts via the cBioPortal for Cancer Genomics (https://www.cbioportal.org): BLCA_MSK_TCGA_2020 (19), BCAN-HCRN-BLCA-2022 (20), MSKCC-SOLIT-BLCA-2014 (21), and MSK-BLCA-2022 (22). Additionally, the IMvigor210CoreBiologies R package (v1.0.0) was used to obtain clinical and molecular data from the IMvigor210 cohort, a phase II trial comprising patients with advanced urothelial carcinoma treated with anti-PD-L1 therapy (23). A unified dataset of clinical, mutational, and survival data was compiled after consolidating these resources. After excluding duplicates and inadequately annotated samples, the final analyzed cohorts included BLCA_MSK_TCGA_2020 (n = 377), MSKCC-SOLIT-BLCA-2014 (n = 109), MSK-BLCA-2022 (n = 1,451), BCAN-HCRN-BLCA-2022 (n = 105), and IMvigor210 (n = 238). To minimize potential confounding, patients who died within two months of treatment initiation or who received antibody-drug conjugates or targeted therapies were excluded. RNA sequencing (RNA-seq) data were available for the IMvigor210 (n = 238) and BCAN-HCRN-BLCA-2022 (n = 89) cohorts. Immune-related gene sets were sourced from the ImmPort database (https://www.immport.org).

### 2.2 Data preprocessing

RNA-seq data from the BCAN-HCRN-BLCA-2022 cohort were preprocessed to ensure data quality. To this end, genes with more than 10% missing values were excluded from the analysis (rowMeans (is. na (data_rna_seq)) ≤ 0.1). Linear interpolation was performed using the zoo R package (v1.8-13) to impute the remaining missing values based on neighboring non-missing data points, with the rule = 2 argument preventing extrapolation beyond the data boundaries.

### 2.3 Somatic mutation analysis

Somatic mutation characteristics were analyzed and visualized using the maftools R package (v2.22.0). The mutational landscape of genes associated with the SWI/SNF chromatin remodeling complex was graphically summarized using OncoPrint plots.

### 2.4 Survival analysis

Kaplan–Meier (KM) survival curves were generated using the survminer R package (v0.5.0) to evaluate the prognostic impact of SWI/SNF complex gene alterations. These curves were used to compare overall survival (OS), progression-free survival (PFS), and disease-free survival (DFS) between patients carrying mutant and wild-type genotypes. Time-dependent receiver operating characteristic (ROC) curves were further constructed using the timeROC package (v0.4) to evaluate the predictive accuracy and sensitivity of mutation-specific prognostic models for OS. For therapeutic response analysis, patients were dichotomized into a Response group, comprising those achieving a Complete Response (CR) or Partial Response (PR), and a No Response group, comprising those with Progressive Disease (PD). This categorization enabled subsequent differential comparisons.

### 2.5 Immune cell infiltration analysis

Immune cell infiltration within the IMvigor210 cohort was assessed using the xCell algorithm through the “IOBR” R package (v0.99.8) to estimate the relative abundance of various immune cell populations. Differences in immune cell infiltration between SWI/SNF mutant and wild-type groups were then systematically analyzed.

### 2.6 Multiplex immunofluorescence staining and image acquisition

Formalin-fixed, paraffin-embedded tissue sections (5 μm thick) from MIBC samples in the Daping cohort (n=13) were subjected to immunofluorescent analysis. Following antigen retrieval with EDTA buffer and blocking with goat serum, sections were further incubated with primary antibodies at 4°C overnight. The primary antibody panel was designed to target: Th1 cells (IFNγ [1:500, Rabbit #ab231036, Abcam] and CD4 [1:600, Rabbit #ab133616, Abcam]), M1 macrophages (CD68 [1:1000, Mouse #ab955, Abcam] and CD86 [1:500, Rabbit #ab317266, Abcam]), regulatory T cells (Tregs, CD4 [1:600, Rabbit #ab133616, Abcam] and FOXP3 [1:100, Rat #13-5773-82, Thermo Fisher Scientific]), and CD8^+^ T cells (CD8α [1:500, Rabbit #D8A8Y, Cell Signaling Technology]). Sections were subsequently incubated with secondary antibodies (RCB054, RecordBio) and labeled with tyramide-conjugated fluorophores according to the manufacturer’s instructions. Images were acquired using an OLYMPUS microscope equipped with Olyvia 4.1 software at 20x magnification, with three random fields captured per sample, and cell expression was quantified using ImageJ software (v1.53t).

### 2.7 Prognostic feature construction using integrated machine learning

We implemented an integrative machine learning framework to construct robust and high-accuracy prognostic models. This framework incorporated eight algorithms and 65 algorithmic combinations (24). The eight algorithms included generalized boosted regression modeling (GBM), elastic net (Enet), ridge regression, stepwise Cox regression, partial least squares regression for Cox (plsRcox), CoxBoost, survival support vector machine (survival-SVM), and supervised principal components (SuperPC).

Model development was conducted independently within the SWI/SNF mutant and wild-type groups following a standardized pipeline:

- Feature Identification: Prognostically relevant features were first identified via univariate Cox regression analysis within the training set. Features with a two-sided p-value < 0.05 were retained for subsequent analysis.
- Feature Refinement: Feature selection was then refined using LASSO regression with 10-fold cross-validation and random survival forests (RSF). The optimal penalty parameter and node impurity reduction guided this process. SHAP values were then calculated, and the top seven genes with the highest predictive impact were selected for final model construction.
- Model Evaluation: All 65 algorithmic combinations were systematically trained and evaluated across both training and validation sets. The best-performing model was primarily selected based on the highest Harrell concordance index (C-index) in the training set and then confirmed on the hold-out validation set.

### 2.8 Statistical analysis

All statistical analyses were conducted using R (v4.4.2) and GraphPad Prism (v10.5.0). Pearson correlation coefficients were calculated to assess linear relationships between genes, with a threshold of 0.8 defined as a strong correlation. Differences in the area under the curve (AUC) values between predictive models were compared using DeLong’s test, implemented with the pROC R package (v1.18.5). Categorical variables were compared using the chi-square test. Continuous variables were compared using either the Wilcoxon rank-sum test or the t-test based on data normality and homoscedasticity. The log-rank (Mantel–Cox) test was used to determine statistical significance between survival curves, with 95% confidence intervals reported. A two-sided p-value < 0.05 was considered statistically significant, with levels annotated as follows: *p < 0.05, **p < 0.01, ***p < 0.001, ****p < 0.0001.

## 3 Results

### 3.1 Frequent alterations in SWI/SNF subunits characterize a high-risk subset of bladder cancer

**Figure 1:**
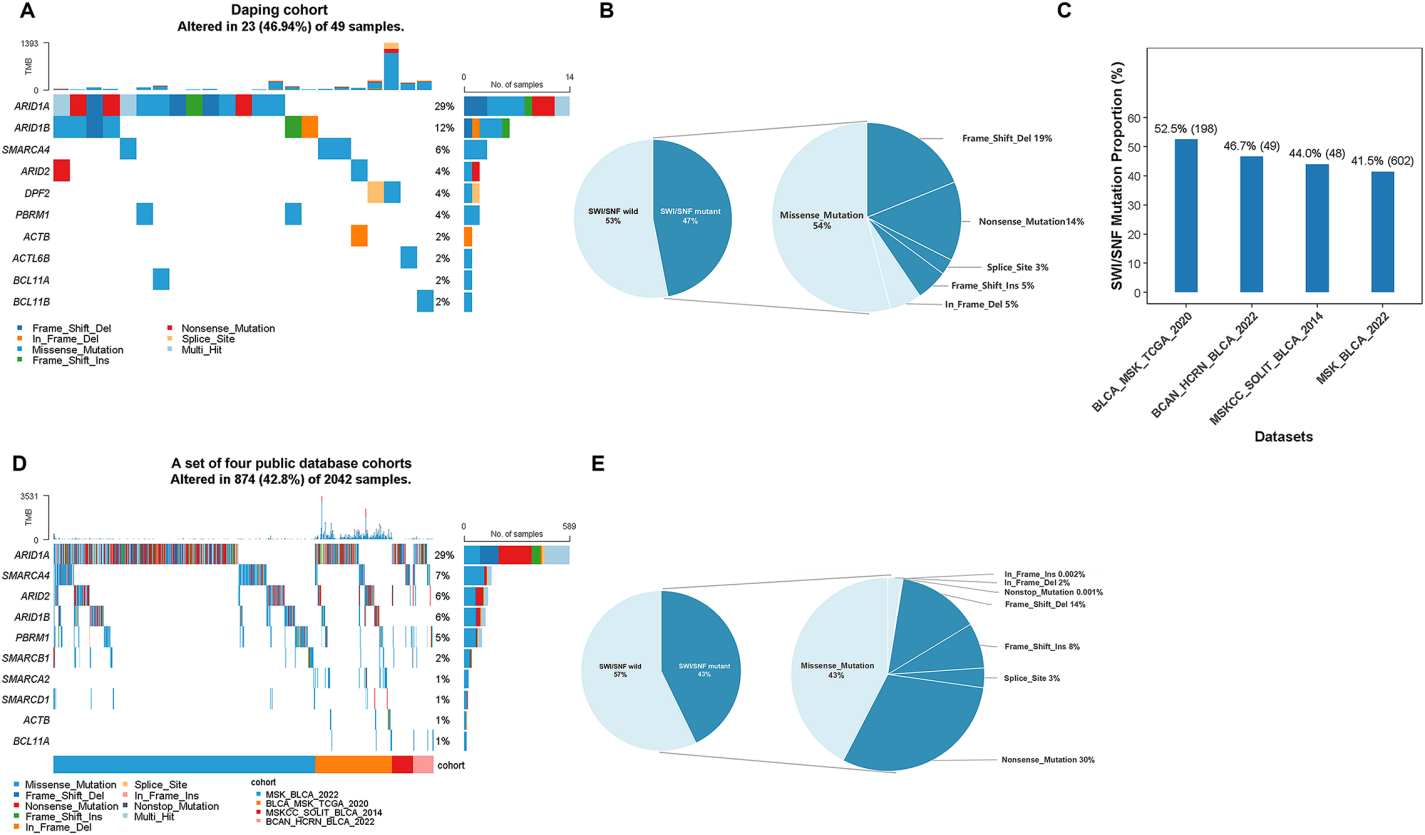
Genomic alterations of SWI/SNF complex genes in bladder urothelial carcinoma (UBC). **(A)** Frequency of mutations in genes encoding the SWI/SNF chromatin remodeling complex among 49 bladder urothelial carcinoma samples from the in-house Daping cohort. **(B)** Distribution of mutation types identified in SWI/SNF complex genes within the Daping cohort. **(C)** Proportion of cases harboring SWI/SNF complex alterations across four independent publicly available UBC cohorts. **(D)** Combined frequency of SWI/SNF gene alterations across the four publicly available cohorts. **(E)** Distribution of alteration types in SWI/SNF complex genes among the four publicly available UBC cohorts.

To define the mutational landscape of SWI/SNF chromatin remodeling complex in UBC, we analyzed the sequencing data from 49 UBC patients in our institutional Daping Cohort. Somatic alterations in at least one of the 29 SWI/SNF subunit genes (hereafter designated SWI/SNF-mutant) were identified in 23 tumors (46.9%; **Figure 1A**). Notably, approximately 41% of these alterations were loss-of-function mutations, including Frame_Shift_Del, Frame_Shift_Ins, Nonsense_Mutation, and Splice_Site (**Figure 1B**). To assess the generalizability of this finding, we further interrogated four independent cohorts: BLCA_MSK_TCGA_2020, BCAN-HCRN-BLCA-2022, MSKCC-SOLIT-BLCA-2014, and MSK-BLCA-2022, encompassing 2,042 patients in total. The prevalence of SWI/SNF alterations was remarkably consistent across these datasets, with rates of 52.5% (198/377) in BLCA_MSK_TCGA_2020, 46.7% (49/105) in BCAN-HCRN-BLCA-2022, 44.0% (48/109) in MSKCC-SOLIT-BLCA-2014, and 41.5% (602/1451) in MSK-BLCA-2022, yielding a pooled mutation rate of 42.8% (**Figures 1C, D**). At the gene level, *ARID1A* exhibited the highest mutation rate (29%), followed by *SMARCA4* (7%), *ARID2* (6%), *ARID1B* (6%), and *PBRM1* (5%). Importantly, 55% of these mutations were predicted to result in loss of function, underscoring their potential as driver events (**Figure 1E**).

We next investigated the clinicopathological correlates of SWI/SNF alterations. Stratified analysis revealed a marked enrichment of these mutations in aggressive disease. Within the Daping cohort, mutation rates were higher in MIBC than in NMIBC tumors (58.8% vs 40.6%; **Supplementary Figure S1A**) and in high-grade versus low-grade neoplasms (51.4% vs 33.3%; **Supplementary Figure S1B**). The same pattern was recapitulated in the public cohorts, with MIBC exhibiting higher mutation rates than NMIBC (50.9% vs 36.2%; *p* = 2 × 10^-5^) and high-grade tumors exceeding low-grade ones (45.9% vs 28.7%; *p* = 3.7 × 10^-3;^**Supplementary Figures S1C, D**). Furthermore, SWI/SNF alterations were also more prevalent in White patients (*p* = 0.012) and in male (*p* = 9.3 × 10^-4^; **Supplementary Figures S1E, F**).

### 3.2 SWI/SNF alterations sensitize bladder cancer to ICB

**Figure 2:**
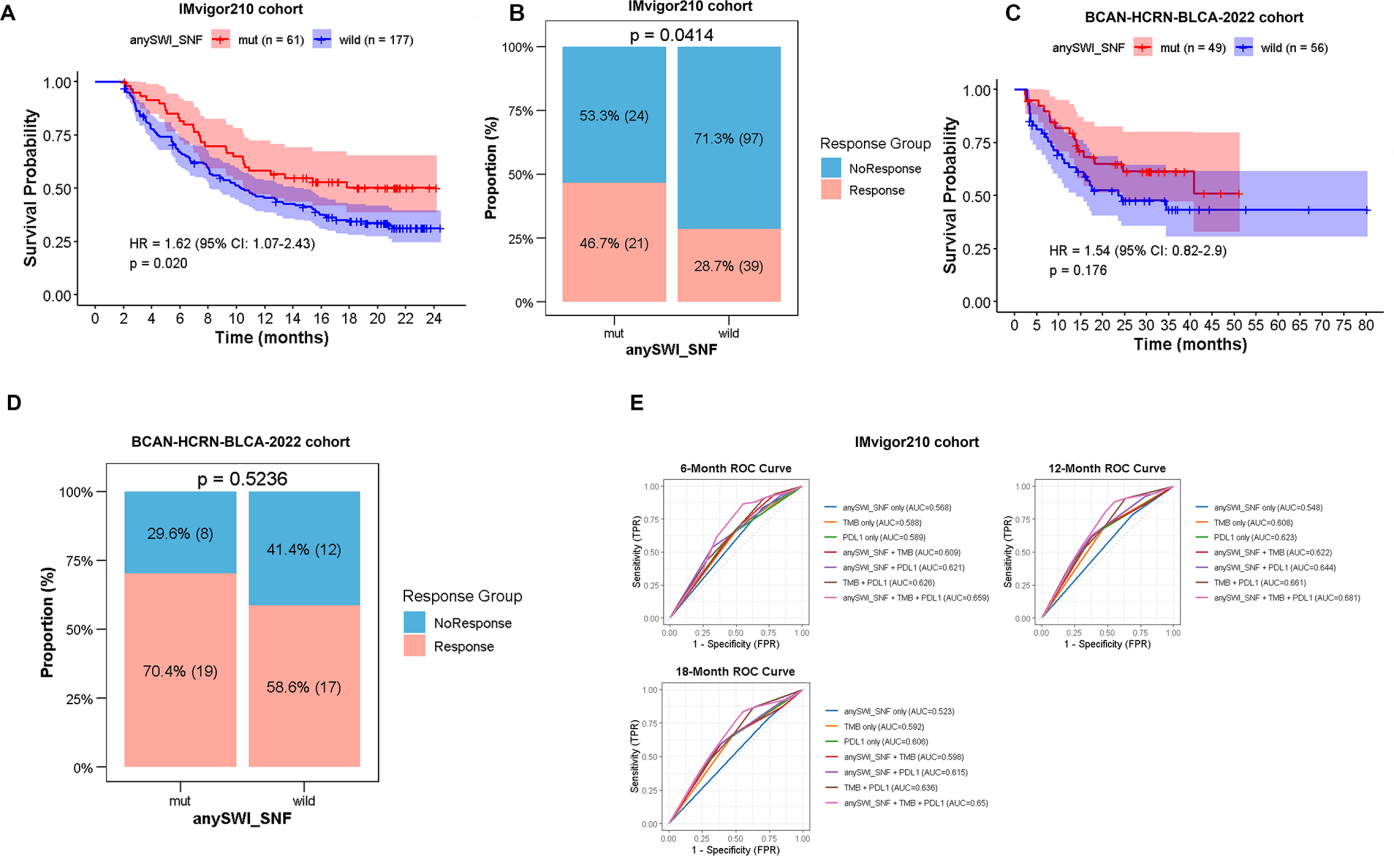
SWI/SNF complex alterations predict improved responses to immunotherapy in bladder cancer. **(A)** Kaplan–Meier curves depicting overall survival following immune checkpoint blockade (ICB) therapy in patients from the IMvigor210 cohort, stratified by SWI/SNF alteration status. **(B)** Proportions of patients achieving objective responses (complete or partial responses) stratified by SWI/SNF alteration status in the IMvigor210. **(C)** Comparison of overall survival in patients with and without SWI/SNF alterations from the BCAN-HCRN-BLCA-2022 cohort. **(D)** Proportions of patients achieving objective responses (complete or partial responses) stratified by SWI/SNF alteration status in the BCAN-HCRN-BLCA-2022 cohorts. **(E)** Comparative predictive performance of SWI/SNF alteration status, both alone and in combination with established biomarkers (TMB and PD-L1), for immunotherapy response.

Despite their association with aggressive clinicopathological phenotypes, SWI/SNF-mutant MIBC patients achieved significantly longer OS in the BLCA-MSK-TCGA-2020 and BLCA-MSKCC-SOLIT-2014 cohorts (*p* = 0.024, HR = 1.49, 95% CI 1.05–2.13; **Supplementary Figures S2A, B**) whereas no significant change was observed in DFS (**Supplementary Figures S2C, D**). This paradox prompted a hypothesis that SWI/SNF alterations confer enhanced sensitivity to systemic therapy. We therefore evaluated the impact of SWI/SNF alterations on immunotherapy outcome in the Imvigor210 and BCAN-HCRN-BLCA-2022 cohorts. Analysis of Imvigor210 cohort (n=238) demonstrated a significantly longer OS (HR = 1.62, 95% CI 1.07–2.43, p = 0.020; **Figure 2A**) and a significantly higher response rate (46.7% vs 28.7%, *p* = 0.041; **Figure 2B**) in patients harboring ≥ 1 SWI/SNF alteration. This trend remained consistent in the smaller BCAN-HCRN-BLCA-2022 cohort (n=105) for both OS (HR = 1.54, 95% CI 0.82–2.9, p = 0.176; **Figure 2C**) and response rate (70.4% vs 58.6%, *p* = 0.5236; **Figure 2D**). To explore the potential mechanism underlying this clinical benefit, transcriptomic analysis was performed, which identified 18 of 28 SWI/SNF genes whose low expression was potentially associated with better outcomes, thereby linking loss-of-function to superior immunotherapy benefit (**Supplementary Figure S2E**).

We further assessed the predictive value of SWI/SNF alterations against established biomarkers. Although SWI/SNF alteration status alone did not outperform PD-L1 expression or TMB, its integration with either or both of these markers consistently augmented the predictive power (**Figure 2E**). Altogether, our results indicate SWI/SNF alterations as a novel marker for identifying a distinct subset of bladder-cancer patients with enhanced sensitivity to ICB.

### 3.3 SWI/SNF alterations enhance tumor immunogenicity and foster an immune-inflamed microenvironment

**Figure 3:**
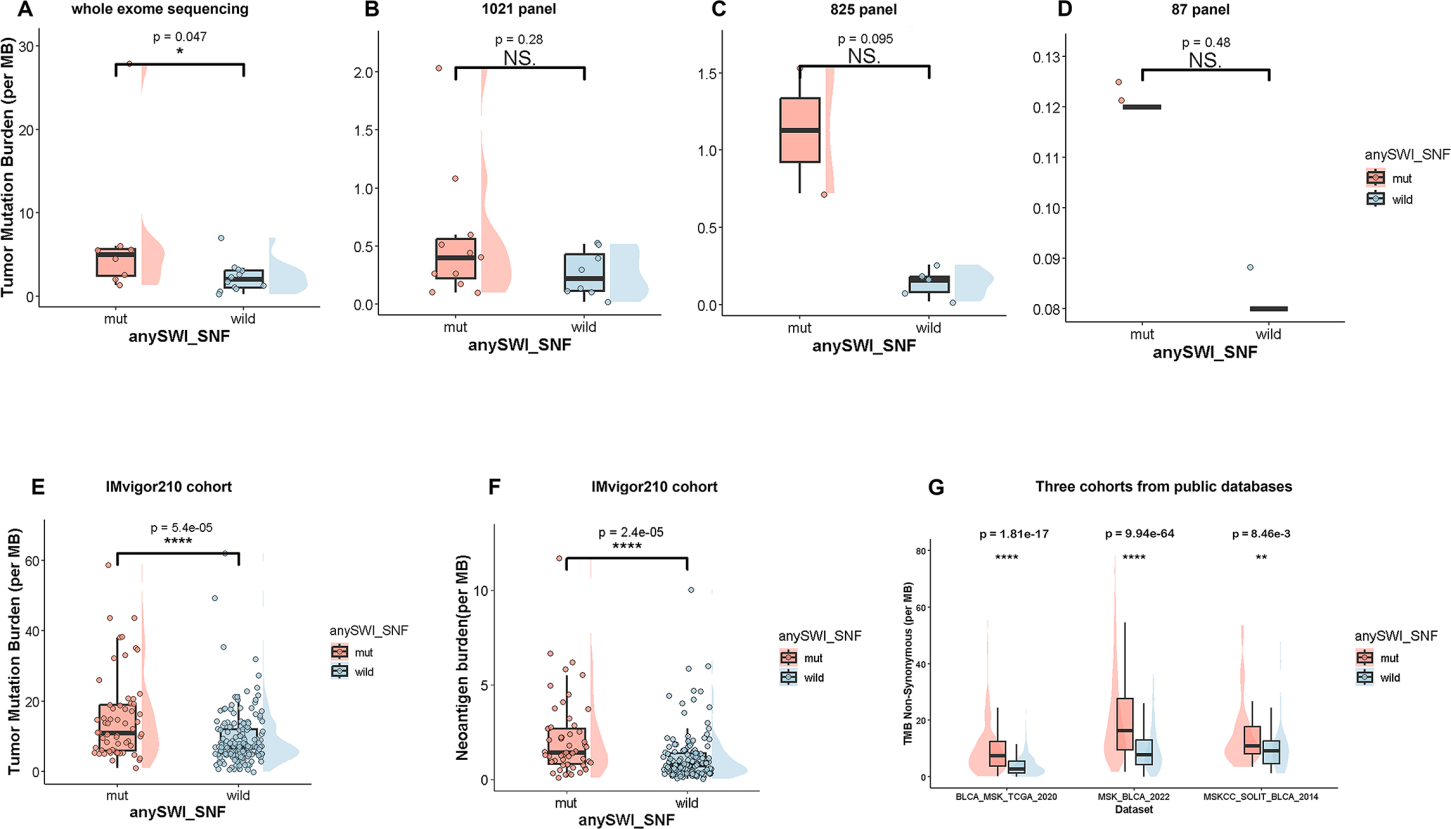
SWI/SNF alterations are associated with increased tumor immunogenicity in bladder cancer. **(A–D)** Box plots showing tumor mutational burden (TMB) in SWI/SNF-mutant bladder tumors compared with wild-type tumors, assessed by and multiple targeted sequencing panels within the Daping cohort. **(E**–**F)** Comparison of TMB and neoantigen burden between SWI/SNF-mutant and wild-type bladder cancer patients from the IMvigor210 cohort. **(G)** Comparison of nonsynonymous mutation load according to SWI/SNF alteration status across three public bladder cancer cohorts. ns, p > 0.05, *p < 0.05, **p < 0.01, ***p < 0.001, ****p < 0.0001.

We posited that the enhanced sensitivity to ICB might stem from increased tumor immunogenicity. Interrogation of multiple cohorts revealed that tumors harboring SWI/SNF alterations displayed markedly higher TMB and neoantigen load. This increase reached statistical significance in the whole-exome sequencing data from our Daping Cohort (*p* = 0.047; **Figures 3A–D**) and was recapitulated in the Imvigor210 (TMB; *p* = 5.4 × 10^-5^; neoantigens: *p* = 2.4 × 10^-5^; **Figures 3E, F**), BLCA-MSK-TCGA-2020 (*p* = 1.81 × 10^-17^), MSK-BLCA-2022 (*p* = 9.94 × 10^-64^), and MSKCC-SOLIT-BLCA-2014 (*p* = 8.46 × 10^-3^; **Figure 3G**). In contrast, the expression levels of key canonical oncogenes (*TP53*, *EGFR*, *PIK3CA*, *FGFR3*) and immune checkpoint molecules (*PD-1*, *PD-L1*, *TIM-3*, *TIGIT*) were comparable between genotypes (**Supplementary Figure S3A**), indicating that the heightened immunogenicity is specifically linked to mutation- and neoantigen-derived antigenicity.

**Figure 4:**
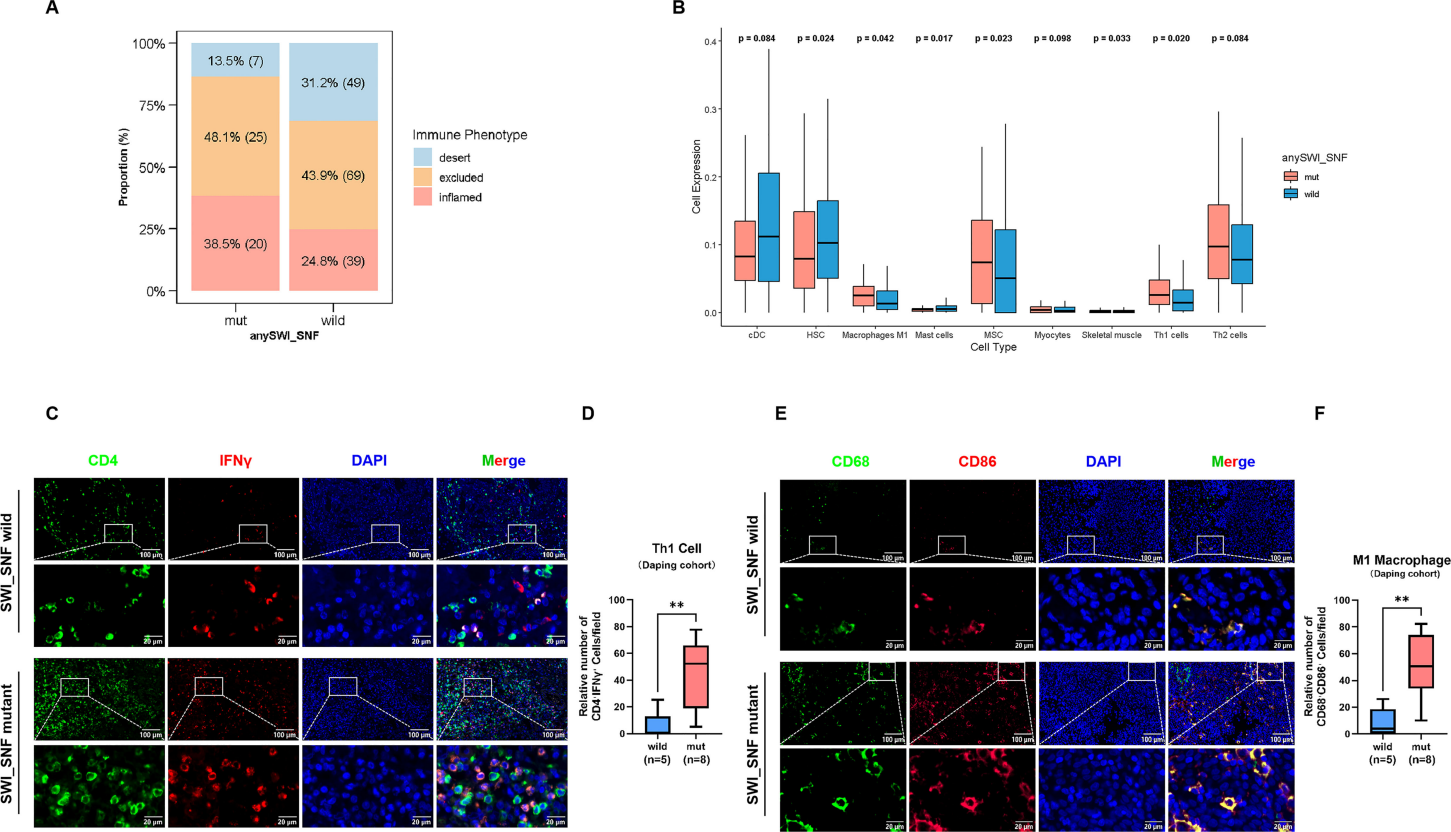
SWI/SNF alterations promote an immune-inflamed tumor microenvironment in bladder cancer. **(A)** Proportion of tumors classified by immune phenotypes according to SWI/SNF alteration status in the IMvigor210 cohort. **(B)** Proportions of tumor-infiltrating immune cell populations in SWI/SNF-mutant and wild-type patients. **(C–F)** Multiplex immunofluorescence staining images and corresponding quantitative analysis of Th1 cells and M1 macrophages infiltration in UBC tissues from the Daping cohort (n = 13). ns, p > 0.05, *p < 0.05, **p < 0.01, ***p < 0.001, ****p < 0.0001.

This elevated antigenicity was accompanied by a shift toward an inflamed tumor microenvironment. In the Imvigor210, SWI/SNF-mutant tumors were more frequently classified as immune-inflamed (38.5% vs 24.8% in wild-type), whereas immune-desert phenotypes were enriched in the wild-type group (31.2% vs 13.5%; **Figure 4A**). Transcriptomic deconvolution of MIBC samples further revealed significantly increased infiltration of M1 macrophages (*p* = 0.042) and Th1 effector T cells (*p* = 0.020), along with a trend towards increased Th2 cells (*p* = 0.084) in mutant tumors (**Figure 4B**, **Supplementary Figure S3B**). These findings were corroborated at the protein level by multiplex immunofluorescence staining within our in-house MIBC cohort (**Figures 4C–F**). We further expanded our multiplex immunofluorescence analysis to encompass additional key immune cell subsets, including CD8^+^ T cells and Tregs. Notably, the infiltration levels of these populations were comparable between SWI/SNF mutant and wild-type tumors, indicating that the mutation-associated immune inflammation is characterized by a specific enrichment of M1 macrophage and helper T cell lineages rather than a global increase in all lymphocyte subsets (**Supplementary Figures S3C–F**). Collectively, our findings link SWI/SNF alterations to an elevated mutational load and an immune-favorable tumor microenvironment, thus establishing a mechanistic basis for the enhanced sensitivity to ICB in this molecular subset.

### 3.4 Genotype-specific prognostic models refine immunotherapy stratification in bladder cancer

**Figure 5:**
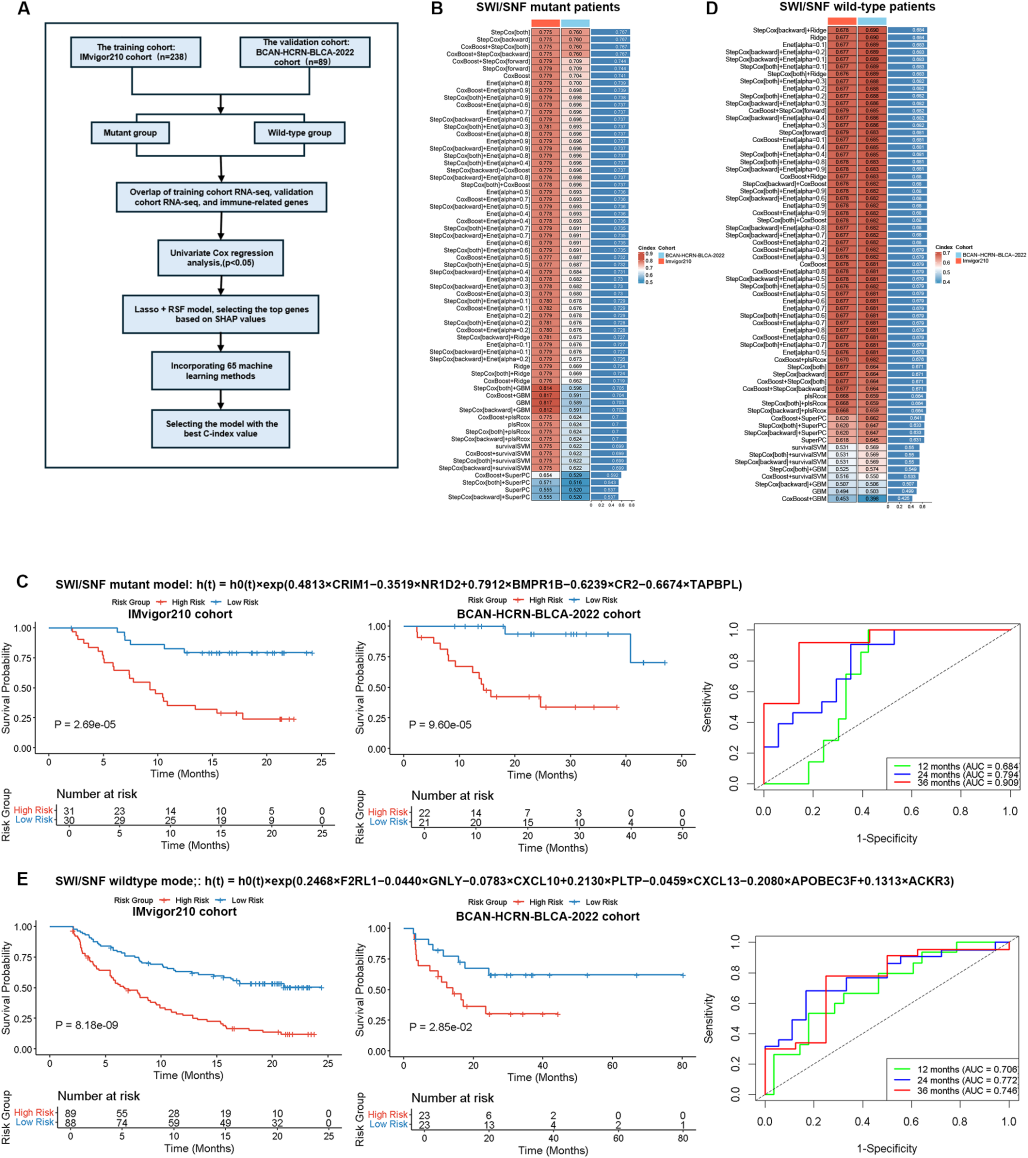
Prognostic modeling based on SWI/SNF alteration-specific transcriptomic signatures in bladder cancer. **(A)** Schematic workflow illustrating the development of SWI/SNF genotype-stratified prognostic models by integrating RNA transcriptomic profiles and alteration status. **(B)** Comparison of concordance indexes (C-indexes) across 65 algorithmic combinations used for developing risk models in the SWI/SNF-mutant patient subgroup from both training and validation cohorts. **(C)** Kaplan–Meier survival analyses comparing overall survival between high- and low-risk patient categories as stratified by genotype-specific prognostic models in both training and validation cohorts within the SWI/SNF-mutant subgroup. **(D)** Comparison of concordance indexes (C-indexes) across 65 algorithmic combinations used for developing risk models in the SWI/SNF wild-type patient subgroups. **(E)** Kaplan–Meier survival analyses comparing overall survival between high- and low-risk patient categories as stratified by genotype-specific prognostic models within the SWI/SNF wild-type subgroup. Time-dependent receiver operating characteristic (ROC) curves are presented at 1-, 2-, and 3-year intervals to assess the predictive accuracy of these models.

Given the significant differences in immune response profiles and microenvironmental infiltration patterns between SWI/SNF mutant and wild-type tumors, as well as substantial heterogeneity within each group, where some mutant tumors show poor ICB response and some wild-type tumors exhibit favorable responses, it is essential to refine patient stratification beyond simple mutation status. To address this, we developed multimodal response-prediction models that integrate SWI/SNF genotype with transcriptomic profiles (**Figure 5A**). Following the exclusion of low-expression genes, we intersected the remaining genes with the validation cohort and an immune gene panel. Univariate Cox regression (*p* < 0.05, **Supplementary Figures S4A, B**, **S5A, B**) identified prognostically relevant features, from which highly collinear features (Pearson *r* > 0.8) were removed. LASSO and Random Survival Forest were used to further refine the signatures, with the most influential genes prioritized by SHAP analysis (**Supplementary Figures S4C–F**, **S5C–F**).

In the SWI/SNF-mutant cohort, a seven-gene signature comprising *CRIM1* (angiogenesis), *VEGFC* (lymphangiogenesis), *BMP7* (tumor microenvironment regulation), *NR1D2* (immune modulation), *BMPR1B* (tumor proliferation), *CR2* (B-cell activation), and *TAPBPL* (antigen presentation) was ultimately retained. A bidirectional stepwise Cox model (StepCox [both]) outperformed the other 64 algorithmic combinations, achieving a mean C-index of 0.767 (training: 0.775, validation: 0.760; **Figure 5B**). The resulting risk score [Risk = exp (0.481 × *CRIM1* – 0.352 × *NR1D2* + 0.791 × *BMPR1B* – 0.624 × *CR2* – 0.667 × *TAPBPL*)] effectively stratified patients into low- and high-risk groups with significantly distinct survival and treatment response in both the Imvigor210 training set (*p* = 2.69 × 10^-5^) and BCAN-HCRN-BLCA-2022 validation set (*p* = 9.6 × 10^-5^). The time-dependent ROC analysis in the validation cohort yielded AUCs of 0.684, 0.794, and 0.909 at 12, 24, and 36 months, respectively, confirming robust predictive accuracy (**Figure 5C**, **Supplementary Figure S4G**).

**Figure 6:**
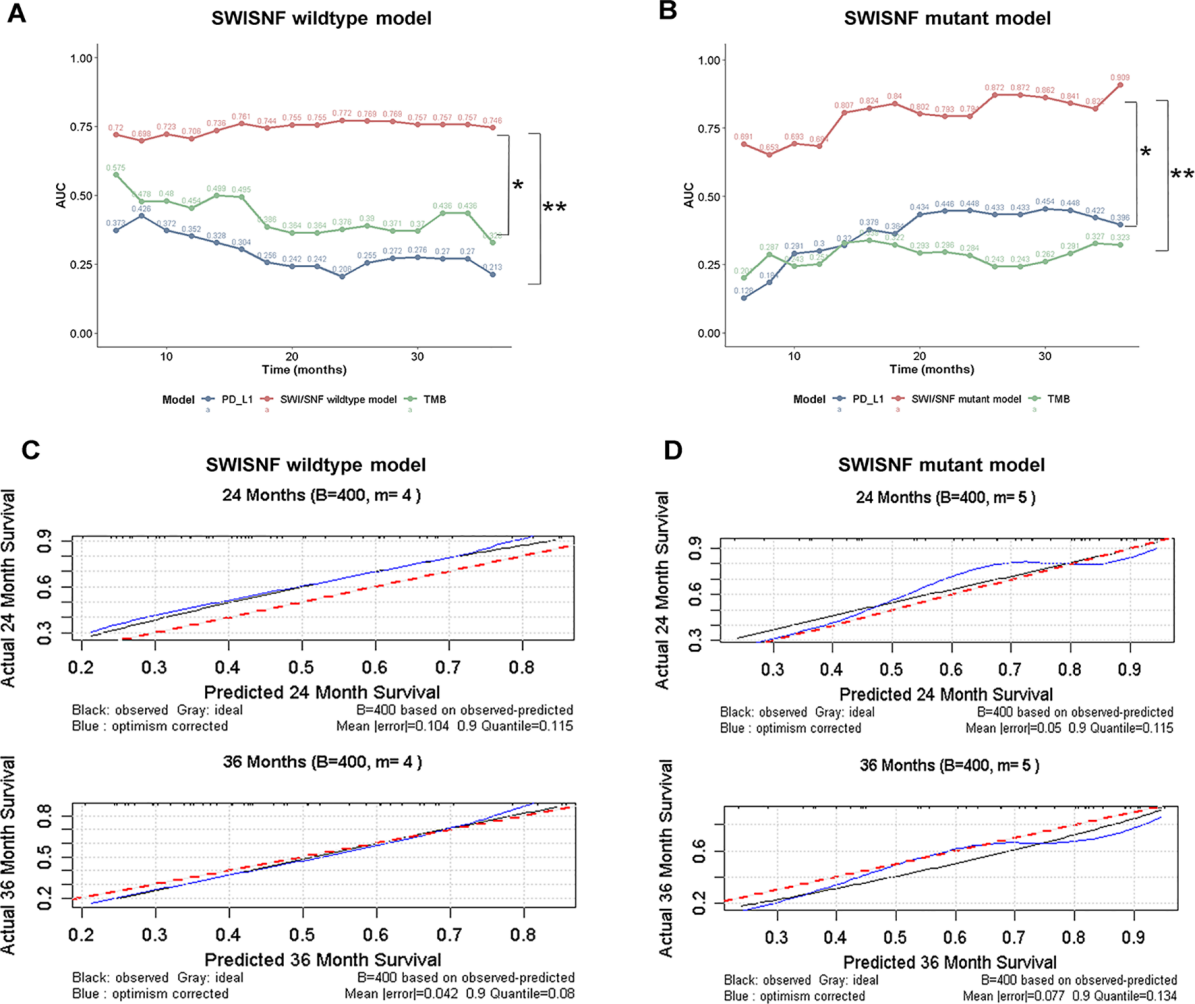
Validation and comparative analysis of genotype-specific prognostic models. **(A, B)** Comparison of time-dependent AUC values between the genotype-specific models and established biomarkers (TMB, PD-L1) within both the SWI/SNF wild-type **(A)** and mutant **(B)** subgroups in the BCAN-HCRN-BLCA-2022 validation cohort. **(C, D)** Calibration curves for the genotype-specific models within both the SWI/SNF wild-type **(C)** and mutant **(D)** subgroups.

In the SWI/SNF wild-type cohort, a distinct set of seven genes was identified as key predictors which comprised *F2RL1* (immune cell recruitment), *GNLY* (immune activation), *CXCL10* (immune cell chemotaxis), *PLTP* (immune modulation), *CXCL13* (lymphocyte recruitment), *APOBEC3F* (mutagenesis), and *ACKR3* (immune cell retention). A hybrid stepwise-backward Cox + Ridge model achieved an average C-index of 0.684 (**Figure 5D**). The corresponding risk score [Risk = exp (0.247 × *F2RL1* – 0.044 × *GNLY* – 0.078 × *CXCL10* + 0.213 × *PLTP* – 0.046 × *CXCL13* – 0.208 × *APOBEC3F* + 0.131 × *ACKR3*)] also successfully stratified patients into low-risk (favorable outcomes) and high-risk groups in both the Imvigor210 training set (*p* = 8.18 × 10^-9^) and BCAN-HCRN-BLCA-2022 validation set (*p* = 0.028). The model exhibited sustained predictive capability, with AUCs of 0.706, 0.772, and 0.746 at 12, 24, and 36 months, respectively (**Figure 5E**, **Supplementary Figure S5G**). Notably, in the BCAN-HCRN-BLCA-2022 validation set, both genotype-specific models demonstrated superior predictive power compared to established biomarkers such as TMB and PD-L1 expression (**Figures 6A, B**), alongside high reliability (**Figures 6C, D**). In summary, we have constructed and validated two genotype-specific prognostic signatures that potentially enable refined risk stratification and guide immunotherapy decisions for both SWI/SNF-mutant and wild-type bladder-cancer patients.

## 4 Discussion

Despite growing recognition of the role of SWI/SNF chromatin-remodeling defects in cancer, their prevalence, functional consequences, and therapeutic implications in UBC have remained poorly characterized. Our study systematically addresses this knowledge gap. We establish SWI/SNF-mutant tumors as a major molecular subtype of UBC, with an aggregate mutation frequency of 42.8% across cohorts, more than double the pan-cancer average reported by Kadoch et al. (19.6%) (12). Beyond their association with aggressive clinicopathological features, SWI/SNF alterations serve as a promising biomarker for superior response to ICB. This enhanced sensitivity is underpinned by a dual mechanism: increased tumor immunogenicity evidenced by elevated TMB and neoantigen load, and a shift towards an immune-inflamed tumor microenvironment characterized by enrichment of M1 macrophages and Th1-polarized T cells. To bridge these biological insights to precision medicine tools, we developed and independently validated genotype-specific prognostic models that integrate SWI/SNF status with immune-stromal transcriptional signatures, proposing a refined tool for risk stratification. Our work lays a rational foundation for biomarker-driven patient selection while paving the way for future therapeutic strategies that leverage chromatin-remodeling deficiencies to enhance immunotherapy efficacy.

SWI/SNF inactivation is one of the most common genetic events in solid tumors yet its consequences are exquisitely context-dependent across solid tumors. In colorectal cancer, truncating mutations in *ARID1A* or *PBRM1* have been linked to increased TMB, robust cytolytic immune infiltration, and durable responses to ICIs (14, 25). By contrast, *PBRM1* loss in renal cell carcinoma is associated with primary ICIs resistance (15), and *ARID1A* deficiency in gastric cancer promotes aggressive metastatic behavior (26). Our study now provides the first comprehensive map linking SWI/SNF subunit disruption to immunotherapy sensitivity in UBC, thereby reconciling these apparent discrepancies. The survival benefit of SWI/SNF alterations reached statistical significance in the IMvigor210 cohort while not in the smaller BCAN-HCRN-BLCA-2022 cohort; this is likely attributable to limitation of the sample size, yet both cohorts exhibited consistent trends towards enhanced immune sensitivity. We demonstrated that loss-of-function lesions across major SWI/SNF subunits converge on a common immune-sensitizing phenotype characterized by heightened neoantigen exposure, enhanced Th1 and M1 polarization, and transcriptional signatures of pre-existing T-cell activation. This mechanistic convergence aligns with recent reports: *ARID1A*-mutant bladder tumors exhibit higher TMB and improved ICIs outcomes, and a significant increase in CD4^+^ T-lymphocyte infiltration has been observed in ICIs responders (27). Collectively, our data establish SWI/SNF alterations as a promising unifying, cross-subunit biomarker of ICIs response in UBC, while larger validation in diverse cohorts might be warranted to fully confirm its utility.

Mechanistically, our data link SWI/SNF loss-of-function to the two established pillars of durable responses to ICB: heightened neoantigen exposure and a pre-existing, Th1-polarized tumor immune microenvironment, providing a clear direction for future validation. Truncating mutations or transcriptional silencing of canonical SWI/SNF subunits are well-known to compromise chromatin accessibility at DNA-damage sites, promoting replication stress, kataegis and a hypermutator phenotype (10). Consistently, *ARID1A* deficiency in melanoma and lung cancer increases cytosolic double-stranded DNA fragments, thereby activating the cGAS-STING pathway and potentiating type-I interferon signaling (28). Similarly, *SMARCA4* deficiency induces widespread intron retention, fostering the generation of neoantigens that enhance MHC-I presentation and elicit T cell-mediated anti-tumor immunity (29). PBRM1 deletion enhances chromosomal accessibility, activates the NF-κB signaling pathway, promotes the secretion of chemokines such as CCL5 and CXCL10, and thereby increases the infiltration of CD8+ T cells and NK cells (25). Our transcriptomic data align with these findings: SWI/SNF-mutant bladder tumors exhibit enriched M1 macrophage and Th1 gene signatures without concomitant up-regulation of *PD-L1* or other immune-inhibitory ligands, supporting an antigen-driven rather than checkpoint-modulated model of immune sensitization.

Given the correlative nature of our findings, these observations provide a compelling foundation for deeper mechanistic investigation. It has not yet fully resolved whether individual SWI/SNF subunits function primarily through cGAS-STING activation, retroelement derepression, or alternative pathways; our findings demonstrated significant correlations but did not establish causality between SWI/SNF loss and UBC development either along or in concert with stromal or immune co-alterations. The observed enrichment of SWI/SNF alterations in male and White patients raises the possibility that germline modifiers or environmental exposures potentially influence the functional consequences, which also underscores the need to ensure generalizability across diverse populations. Future studies employing CRISPR perturbation screens, lineage-specific conditional knockouts, and single-cell multi-omics will be needed to delineate the precise molecular circuitry by which SWI/SNF disruption licenses anti-tumor immunity in bladder cancer. Complementary validation in multi-ethnic cohorts will also be needed to confirm utility of this biomarker in underrepresented populations.

A major contribution of our work lies in the development of genotype-specific, multi-omic risk models that translate SWI/SNF biology into clinically applicable decision tools. Previous attempts to predict ICIs benefit in bladder cancer have predominantly relied on single-modality biomarkers, including PD-L1 CPS ≥ 10 (as defined in IMvigor210) (9), and TMB ≥ 10 mut/Mb (as used in CheckMate-275) (8), to predict durable clinical benefit beyond six months. All these efforts yielded modest AUCs (TMB 12-month AUC: 0.601; PD-L1 12-month AUC: 0.646) in the validation set (30). In this study, we explicitly stratified patients (from the same cohort analyzed by Yarchoan M, et al.) by SWI/SNF alteration status and integrated immune-related transcriptomic features, thereby leveraging a biologically informed interaction that substantially enhances prognostic precision. In SWI/SNF-mutant tumors, a parsimonious five-gene signature achieved time-dependent AUCs of 0.684, 0.794, 0.909 at 12, 24, 36 months, respectively, while an orthogonal seven-gene panel achieved AUCs of 0.706, 0.772, 0.746 in the wild-type context. Both models demonstrated marked superiority over traditional models employing TMB or PD-L1 in the validation cohort.

Importantly, both models were derived through a stringent machine-learning pipeline that incorporated Univariate Cox Regression, LASSO regularization, Random Survival Forest, and SHAP-based interpretability analysis, thereby minimizing overfitting while preserving biological relevance. We further evaluated the prognostic relevance of the model genes in the context of immunotherapy and found that the majority were significantly associated with OS following ICIs (**Supplementary Figure S6A**), which aligns with their previously reported roles in immune response (31–39). Corroborating their biological plausibility, stratification of samples by expression levels of these genes revealed clear correlations with immune contexture. For instance, elevated expressions of *CXCL10, CXCL13, GNLY, APOBEC3F*, and *TAPBPL* were associated with higher immune scores and lower stromal scores, reflecting an immune-inflamed microenvironment. Moreover, several genes, including *CR2, CXCL10, CXCL13, GNLY, APOBEC3F*, and *TAPBPL*, were found to be correlated positively with CD8^+^ T-cell infiltration, whereas *ACKR3, CRIM1, BMPR1B*, and *PLTP* were found to be linked to increased fibroblast abundance (**Supplementary Figure S6B**). This concordance between computational prediction and immunobiological function profoundly reinforces the interpretability and mechanistic grounding of our model signatures.

Practically, the resulting risk scores can be computed from standard bulk RNA-seq or targeted NGS assays already available in many centers, potentially offering a seamless path to prospective validation. Ultimately, these tools could enable adaptive, biomarker-driven clinical trials that direct SWI/SNF-mutant patients to first-line ICIs while reserving intensified combination regimens for those with high-risk, wild-type tumors.

## 5 Conclusion

Notwithstanding the retrospective nature of the cohorts and lack of direct mechanistic interrogation, these limitations do not undermine the immediate translational value of our findings. The high prevalence of SWI/SNF alterations is consistently observed across datasets. Coupled with their reproducible association with enhanced response to ICIs, this provides a compelling signal that merits rapid evaluation in prospective trials. Furthermore, while the precise molecular circuitry linking SWI/SNF loss to enhanced antigen presentation and T-cell infiltration remains to be fully elucidated, our integrated prognostic models already outperform existing single-modality biomarkers and can be deployed with standard RNA-seq and targeted NGS assays already available in most centers. In short, this work establishes SWI/SNF alteration status as a clinically actionable anchor for personalizing immunotherapy in MIBC and lays the groundwork for future mechanistic studies and prospective validation.

## Data Availability

The datasets presented in this study can be found in online repositories. The names of the repository/repositories and accession number(s) can be found below: https://ngdc.cncb.ac.cn/search/specific?db=hra&q=HRA004401, HRA004401.

## Supplementary material

Supplementary Figure 1 Distribution of SWI/SNF alterations across clinicopathological subgroups in bladder cancer. **(A, B)** Proportion of SWI/SNF alterations in muscle-invasive bladder cancer (MIBC) versus non-muscle-invasive bladder cancer (NMIBC) and high-grade versus low-grade tumor subtypes within the Daping cohort. **(C, D)** Validation of mutation distribution findings in a public cohort by comparing MIBC versus NMIBC tumors and high-grade versus low-grade tumors. **(E, F)** Comparison of SWI/SNF alteration frequencies according to sex and race in the public cohort.

Supplementary Figure 2 Prognostic impact of SWI/SNF alterations. **(A, B)** Kaplan–Meier survival curves showing overall survival among SWI/SNF-mutant compared with wild-type MIBC patients in the BLCA-MSK-TCGA-2020 and BLCA-MSKCC-SOLIT-2014 cohorts. **(C, D)** Kaplan–Meier curves indicating disease-free survival between SWI/SNF-mutant and wild-type patients in these same cohorts. **(E)** Transcriptomic analysis of the correlation between SWI/SNF gene expression and outcomes following immunotherapy. ns, p > 0.05, *p < 0.05, **p < 0.01, ***p < 0.001, ****p < 0.0001.

Supplementary Figure 3 Transcriptomic and immune infiltration characterization according to SWI/SNF alteration status. **(A)** Comparison of mRNA expression levels of canonical oncogenes (TP53, EGFR, PIK3CA, FGFR3) and immune checkpoint molecules (PD-1, CD274, TIM-3, TIGIT) between SWI/SNF-mutant and wild-type tumors across cohorts. **(B)** Multiple-group box plot illustrating proportions of tumor-infiltrating immune cell types between SWI/SNF-mutant and wild-type bladder cancer patients. **(C-F)** Multiplex immunofluorescence staining images and corresponding quantitative analysis of CD8+ T cells or Treg cells infiltration in UBC tissues from the Daping cohort (n = 13). ns, p > 0.05, *p < 0.05, **p < 0.01, ***p < 0.001, ****p < 0.0001.

Supplementary Figure 4 Feature selection and prognostic model construction to predict immunotherapy responses in the SWI/SNF-mutant subgroup. **(A)** Venn diagram illustrating the intersection of candidate genes derived from SWI/SNF-mutant samples within the training cohort, validation cohort, and immune gene panel. **(B)** Forest plot showing genes significantly associated with survival (P < 0.05) as determined by univariate Cox regression analysis. **(C**–**D)** Variable screening using Lasso regression analysis. **(E**–**F)** Random survival forest analysis employing SHAP values to rank candidate genes based on their predictive importance for survival outcomes. **(G)** Stacked bar plot showing proportions of immunotherapy responses stratified by prognostic model-derived risk scores in the SWI/SNF-mutant subgroup.

Supplementary Figure 5 Feature selection and prognostic model construction to predict immunotherapy responses in the SWI/SNF-wild-type subgroup. **(A)** Venn diagram showing the overlap of candidate genes identified in SWI/SNF-wild-type samples across the training cohort, validation cohort, and immune gene panel. **(B)** Forest plot displaying genes significantly associated with survival (P < 0.05) as determined by univariate Cox regression analysis. **(C, D)** Variable screening performed using Lasso regression analysis. **(E, F)** Random survival forest analysis using SHAP values to rank gene importance of genes for survival. **(G)** Stacked bar plot illustrating the proportions of immunotherapy responses stratified by risk scores derived from the prognostic model in the SWI/SNF-wild-type subgroup.

Supplementary Figure 6 Associations of Selected Genes with Prognosis and Immune Microenvironment. **(A)** Prognostic relevance of the model genes for overall survival following immunotherapy. **(B)** Heatmap visualizing differences in immune cell infiltration and stromal abundance between high- and low-expression groups of the model-derived genes.
