# Supplementary figures and images for: SWI/SNF complex alterations predict immunotherapy response in bladder cancer
Source: Front Immunol. 2025 Dec 8;16:1708324. doi: 10.3389/fimmu.2025.1708324 (PMC12719508; doi:10.3389/fimmu.2025.1708324)

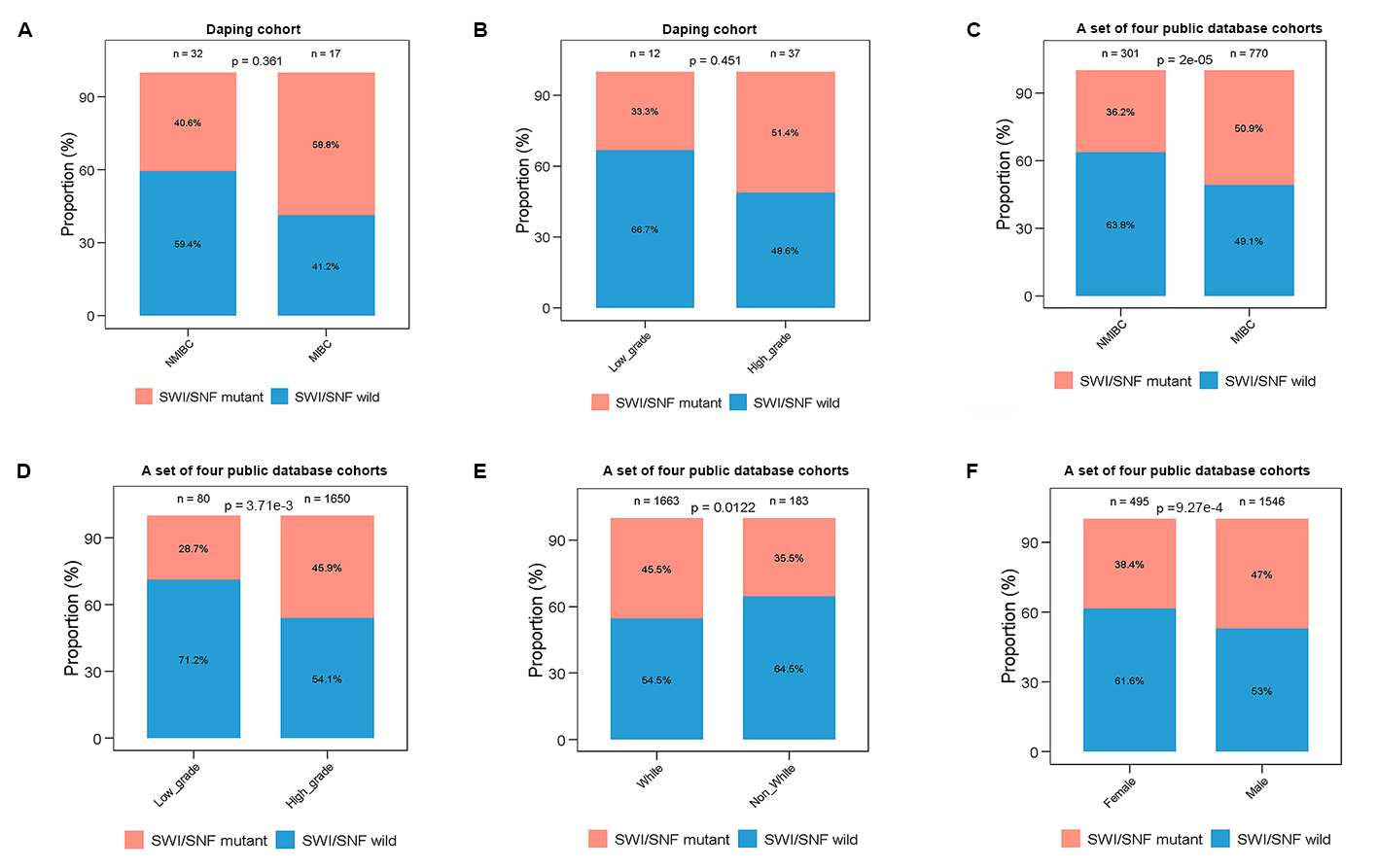

Supplement: Supplementary Figure 1 — Distribution of SWI/SNF alterations across clinicopathological subgroups in bladder cancer. (A, B) Proportion of SWI/SNF alterations in muscle-invasive bladder cancer (MIBC) versus non-muscle-invasive bladder cancer (NMIBC) and high-grade versus low-grade tumor subtypes within the Daping cohort. (C, D) Validation of mutation distribution findings in a public cohort by comparing MIBC versus NMIBC tumors and high-grade versus low-grade tumors. (E, F) Comparison of SWI/SNF alteration frequencies according to sex and race in the public cohort. [file Image1.tif]

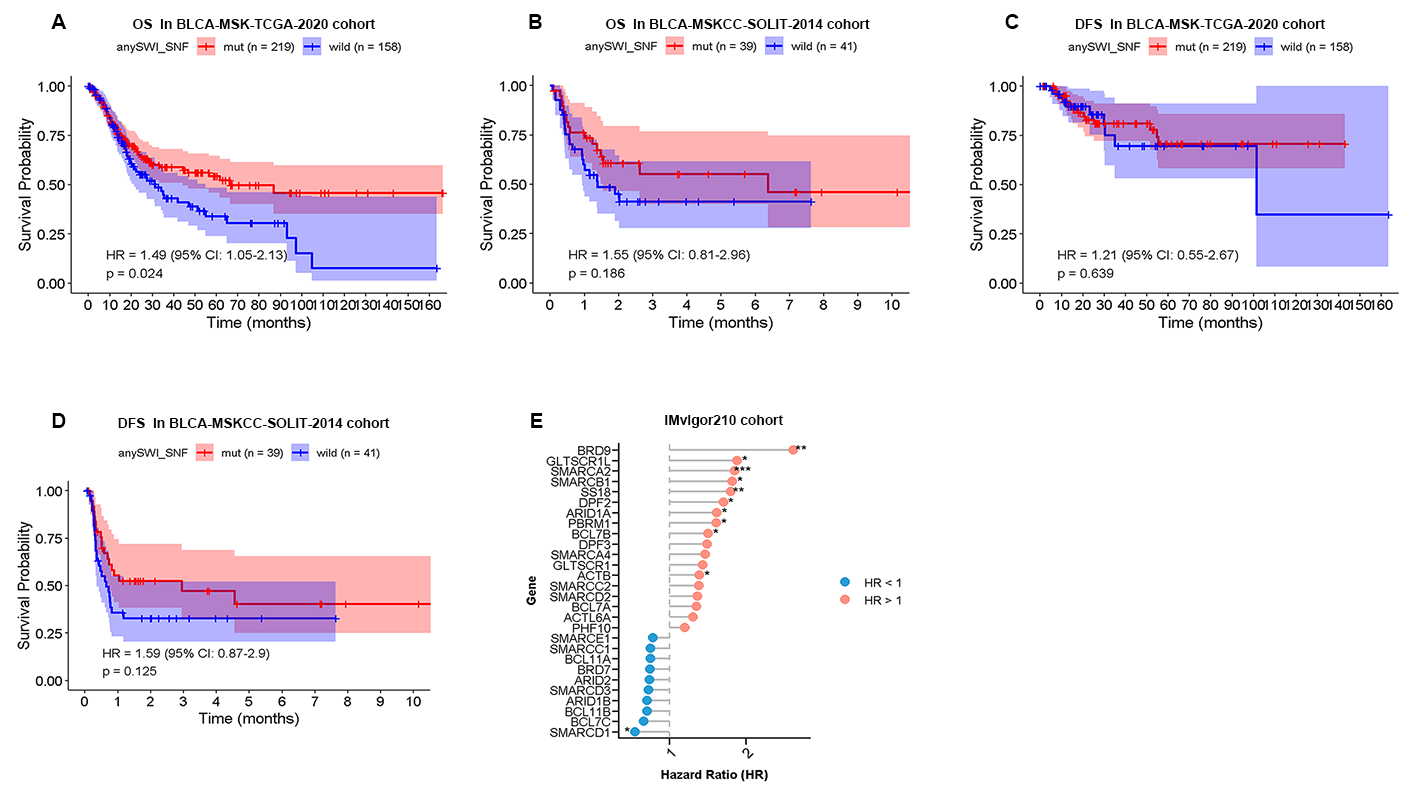

Supplement: Supplementary Figure 2 — Prognostic impact of SWI/SNF alterations. (A, B) Kaplan–Meier survival curves showing overall survival among SWI/SNF-mutant compared with wild-type MIBC patients in the BLCA-MSK-TCGA-2020 and BLCA-MSKCC-SOLIT-2014 cohorts. (C, D) Kaplan–Meier curves indicating disease-free survival between SWI/SNF-mutant and wild-type patients in these same cohorts. (E) Transcriptomic analysis of the correlation between SWI/SNF gene expression and outcomes following immunotherapy. ns, p > 0.05, *p < 0.05, **p < 0.01, ***p < 0.001, ****p < 0.0001. [file Image2.tif]

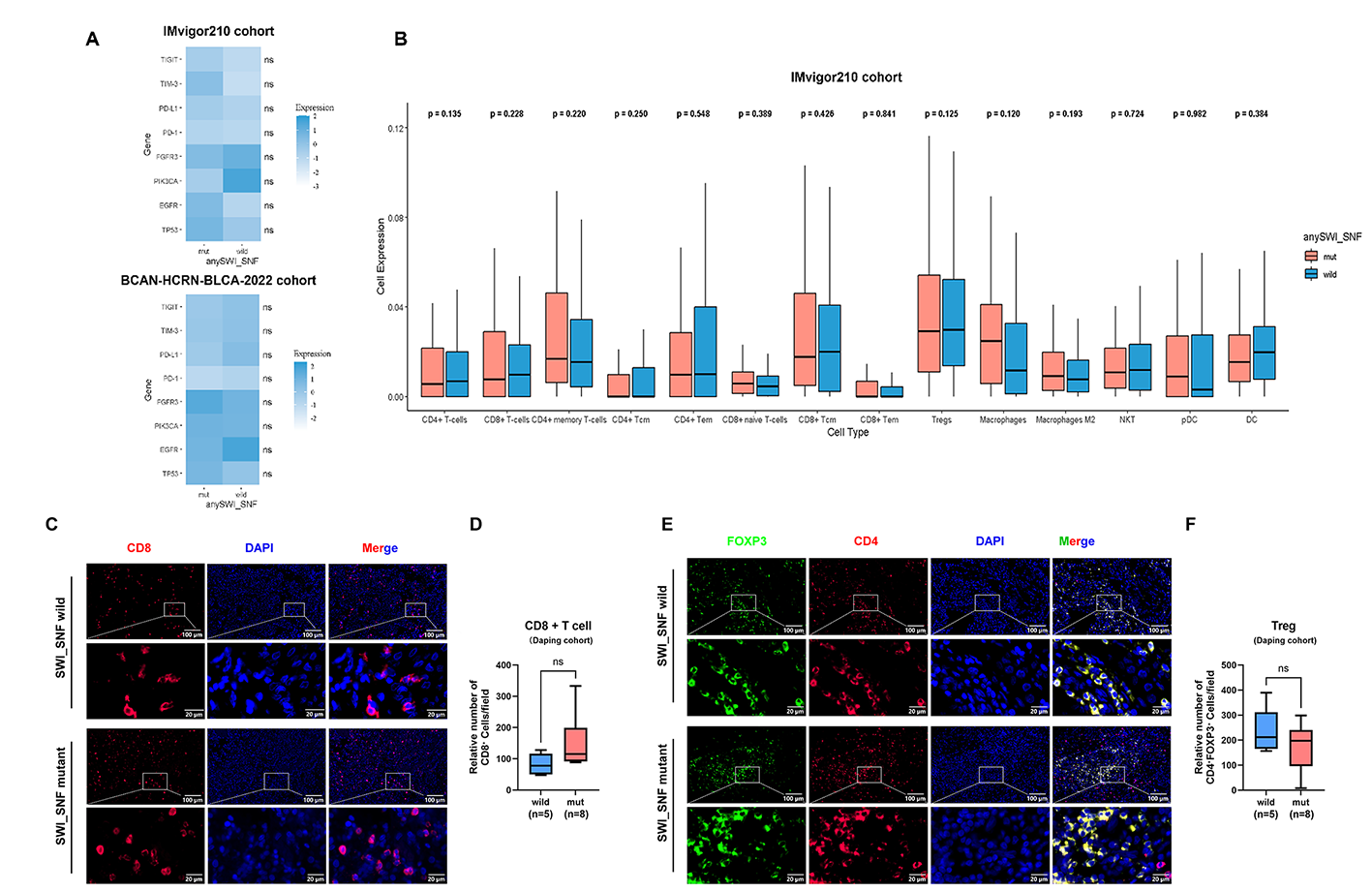

Supplement: Supplementary Figure 3 — Transcriptomic and immune infiltration characterization according to SWI/SNF alteration status. (A) Comparison of mRNA expression levels of canonical oncogenes (TP53, EGFR, PIK3CA, FGFR3) and immune checkpoint molecules (PD-1, CD274, TIM-3, TIGIT) between SWI/SNF-mutant and wild-type tumors across cohorts. (B) Multiple-group box plot illustrating proportions of tumor-infiltrating immune cell types between SWI/SNF-mutant and wild-type bladder cancer patients. (C-F) Multiplex immunofluorescence staining images and corresponding quantitative analysis of CD8+ T cells or Treg cells infiltration in UBC tissues from the Daping cohort (n = 13). ns, p > 0.05, *p < 0.05, **p < 0.01, ***p < 0.001, ****p < 0.0001. [file Image3.tif]

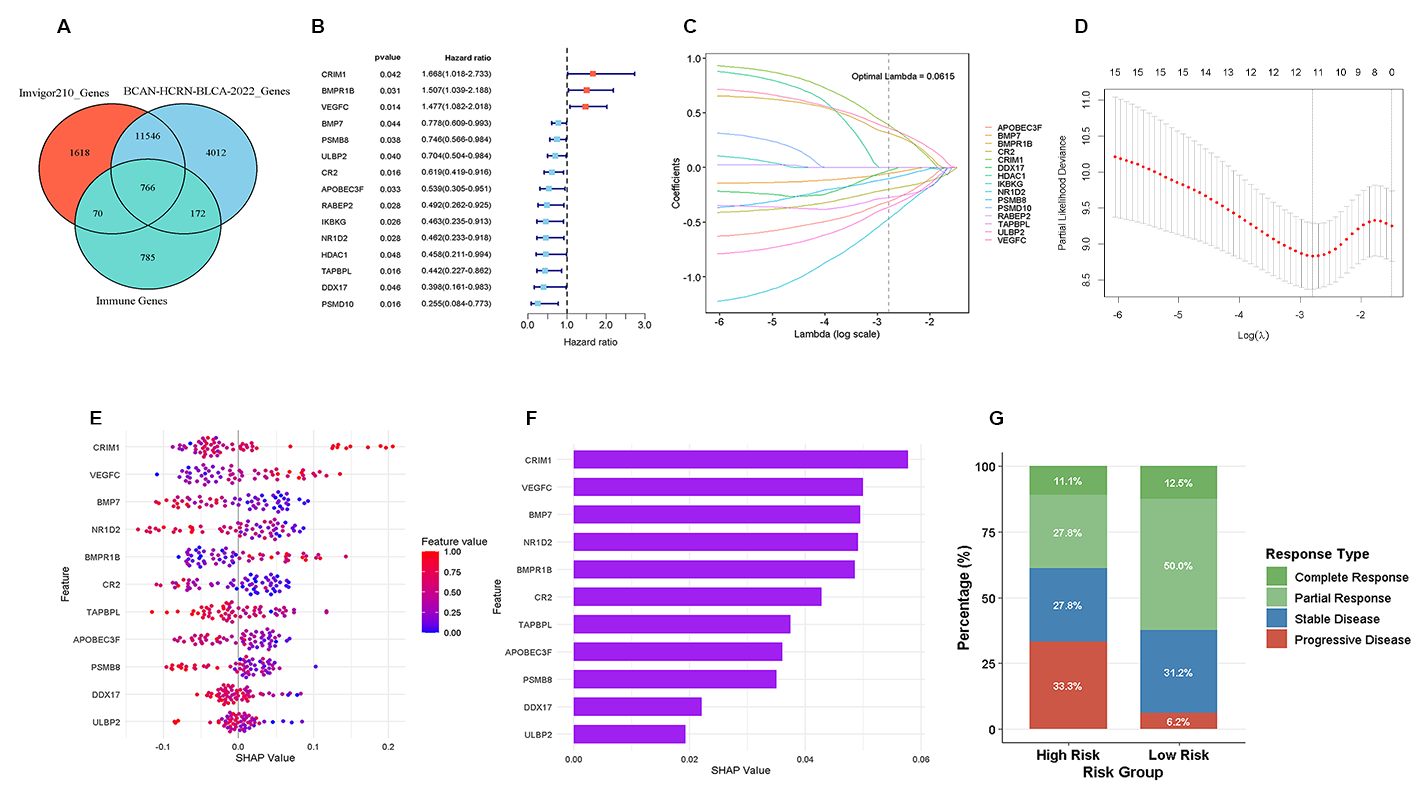

Supplement: Supplementary Figure 4 — Feature selection and prognostic model construction to predict immunotherapy responses in the SWI/SNF-mutant subgroup. (A) Venn diagram illustrating the intersection of candidate genes derived from SWI/SNF-mutant samples within the training cohort, validation cohort, and immune gene panel. (B) Forest plot showing genes significantly associated with survival (P < 0.05) as determined by univariate Cox regression analysis. (C–D) Variable screening using Lasso regression analysis. (E–F) Random survival forest analysis employing SHAP values to rank candidate genes based on their predictive importance for survival outcomes. (G) Stacked bar plot showing proportions of immunotherapy responses stratified by prognostic model-derived risk scores in the SWI/SNF-mutant subgroup. [file Image4.tif]

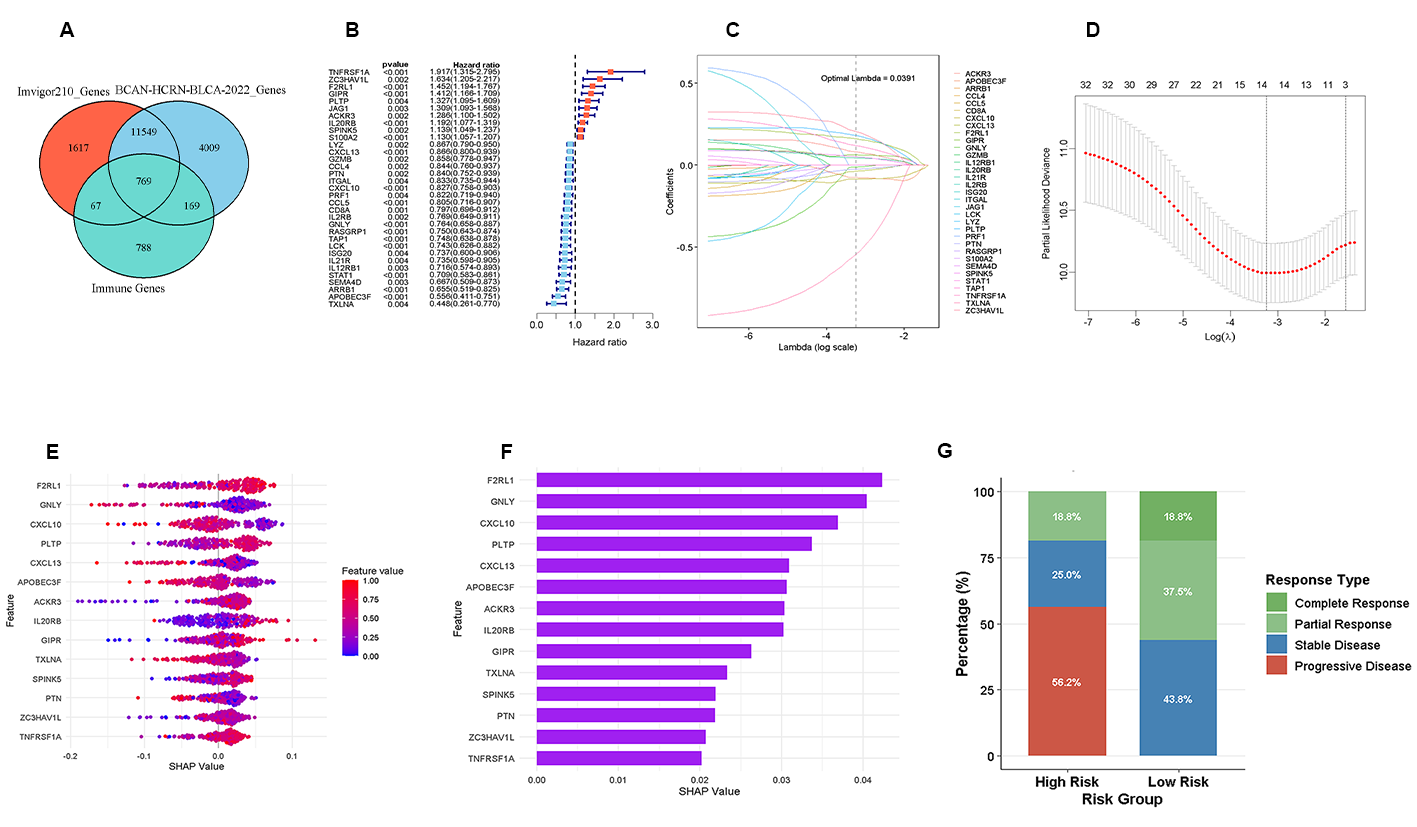

Supplement: Supplementary Figure 5 — Feature selection and prognostic model construction to predict immunotherapy responses in the SWI/SNF-wild-type subgroup. (A) Venn diagram showing the overlap of candidate genes identified in SWI/SNF-wild-type samples across the training cohort, validation cohort, and immune gene panel. (B) Forest plot displaying genes significantly associated with survival (P < 0.05) as determined by univariate Cox regression analysis. (C, D) Variable screening performed using Lasso regression analysis. (E, F) Random survival forest analysis using SHAP values to rank gene importance of genes for survival. (G) Stacked bar plot illustrating the proportions of immunotherapy responses stratified by risk scores derived from the prognostic model in the SWI/SNF-wild-type subgroup. [file Image5.tif]

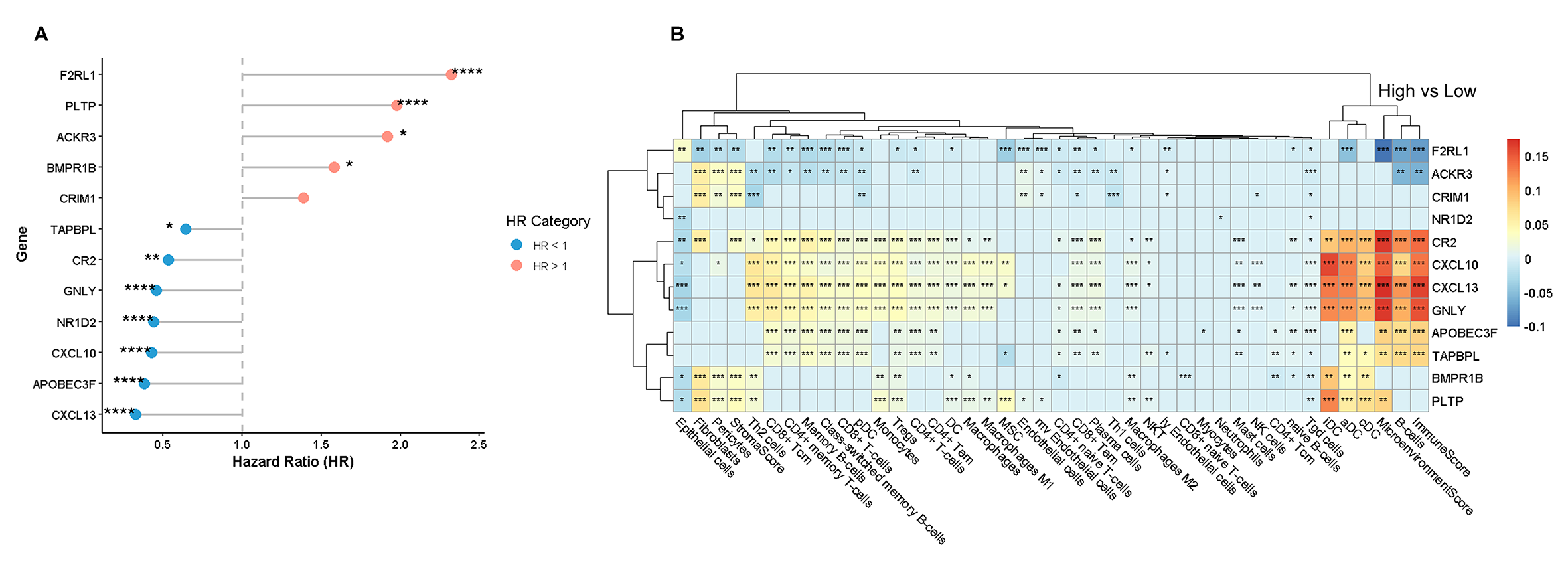

Supplement: Supplementary Figure 6 — Associations of Selected Genes with Prognosis and Immune Microenvironment. (A) Prognostic relevance of the model genes for overall survival following immunotherapy. (B) Heatmap visualizing differences in immune cell infiltration and stromal abundance between high- and low-expression groups of the model-derived genes. [file Image6.tif]
